# A Comprehensive Assessment of Prescription Patterns for Anemia Management in Pregnant Women: A Study From Anand District, India

**DOI:** 10.7759/cureus.58952

**Published:** 2024-04-24

**Authors:** Musaratafrin Saiyed, Jalpa V Suthar

**Affiliations:** 1 Department of Clinical Pharmacology, Ramanbhai Patel College of Pharmacy, Charotar University of Science and Technology (CHARUSAT), Anand, IND; 2 Clinical Pharmacology, Ramanbhai Patel College of Pharmacy, Charotar University of Science and Technology (CHARUSAT), Anand, IND

**Keywords:** prevalence, iron supplements, pregnancy, anemia, prescribing patterns

## Abstract

Introduction

Pregnancy induces various physiological changes, often leading to complications. Physiological anemia of pregnancy, resulting from increased plasma volume and erythropoietin levels, poses significant health risks. Adverse outcomes associated with anemia during pregnancy include maternal and perinatal mortality, premature delivery, and low birth weight. Drug utilization research aims to promote rational drug use for improving health outcomes. The Food and Drug Administration (FDA) categorizes drugs based on teratogenic risk, providing guidance for clinicians. This study aims to analyze prescription trends and FDA risk categories for anemia in pregnant women in the Anand district.

Materials and methods

The study received institutional ethics approval and involved 816 pregnant women attending antenatal clinics from December 2021 to March 2023. Participants provided informed consent, and data collection included hemoglobin (Hb) levels at each trimester, categorizing participants into anemic (Hb < 11 gm/dL) and non-anemic groups. Prescribed drugs were recorded, and their essentiality was assessed using the WHO Essential Medicines List (WHO-EML) and the National List of Essential Medicines-2022 (NLEM-2022). FDA drug risk categories were utilized for assessing drug safety. Descriptive and statistical analyses were performed.

Results

Anemia prevalence across trimesters ranged from 62.50% to 65.93%, with an overall average of 64.42%. Iron and folic acid supplementation were significant across trimesters, with varying rates of prescription. Calcium supplementation showed fluctuations, with 100% prescription rates in later trimesters. Ascorbic acid was significantly prescribed in anemic pregnant women throughout pregnancy. Multivitamins were consistently prescribed, emphasizing their importance. The WHO-EML and NLEM-2022 highlighted essential micronutrients, while FDA categories indicated drug safety.

Conclusion

Anemia prevalence remained high throughout pregnancy, emphasizing the need for consistent supplementation. Prescription patterns aligned with evidence-based guidelines, focusing on iron and folic acid supplementation. Variations in calcium prescription suggest trimester-specific considerations. Prescription trends reflect a responsible approach to managing anemia during pregnancy, emphasizing prophylactic iron and folic acid therapy. The absence of high-risk medications underscores cautious prescribing practices. This study contributes valuable insights into evidence-based pharmacotherapy and maternal health care.

## Introduction

Pregnancy is associated with a wide range of pharmacokinetic and physiological changes. These changes quite often present physiological complications during pregnancy [1]. During pregnancy, the body undergoes several physiological changes, including an expansion of plasma volume and an increase in erythropoietin levels. These changes are adaptive mechanisms to support the growth of the fetus, oxygen transport, and uterine blood flow. The expansion of plasma volume during pregnancy leads to hemodilution and a decrease in the hematocrit level, resulting in a condition known as physiological anemia of pregnancy [2]. The prevalence of anemia in pregnant women globally ranges from 30% to 75%, with the highest rates observed in developing countries [3]. In India, the National Family Health Survey-5 (NFHS-5) reports the prevalence of anemia in pregnant women as 42.6% [4]. Anemia in pregnancy is associated with increased rates of maternal and perinatal mortality, premature delivery, low birth weight, and other adverse outcomes [5].

Drug utilization research was defined by the World Health Organization (WHO) as “the marketing, distribution, prescription, and use of drugs in a society, with special emphasis on the resulting medical, social, and economic consequences” [6]. The principal aim of drug utilization research is to facilitate the rational use of drugs in the population, thereby improving health outcomes. Drug utilization pattern studies were conducted to promote and increase rational drug therapy and also used to evaluate the prescribing pattern and lifestyle modification followed in anemic pregnant women [7]. In addition, essential drugs satisfy the healthcare needs of the major population. In 1979, the Food and Drug Administration (FDA) developed a system determining the teratogenic risk of drugs by considering the quality of data from animal and human studies. It provides therapeutic guidance for the clinician. Category A is considered the safest category but some drugs from categories B, C, and D are also used during pregnancy. Category X is the only rating that denotes a drug is absolutely contraindicated for use during pregnancy [8].

This study was undertaken to conduct a full comprehensive analysis of prescription trends and FDA risk categories for anemia management in pregnant women of Anand district, India. A random sampling technique was used to make sure that each pregnant woman had an equal chance of being selected for participation. This approach was selected to minimize bias and ensure the representativeness of our sample population.

## Materials and methods

**Figure 1:**
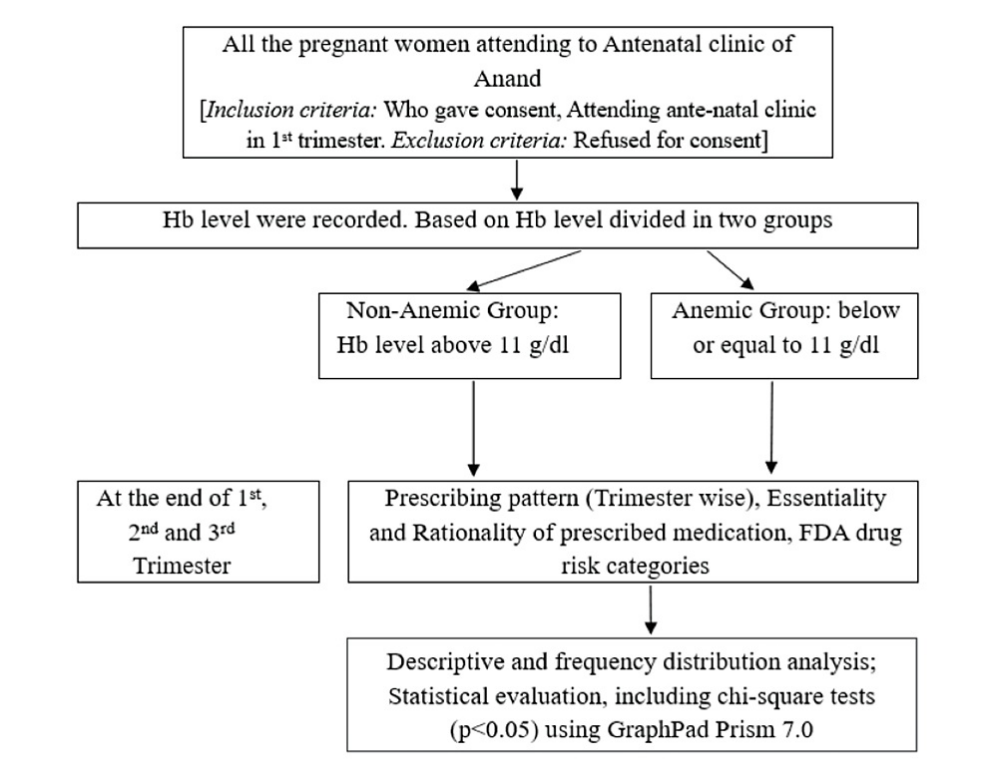
Study design flow chart Hb: hemoglobin.

The study protocol, identified as CHA/IEC/ADM/21/11/1643.02, received ethical approval on November 30, 2021, from the Institutional Ethics Committee (IEC), Charotar University of Science and Technology (CHARUSAT), Changa, Anand. This research comprised a prospective, observational study conducted over 15 months, spanning from December 2021 to March 2023, with a specific focus on 816 pregnant women (Figure 1).

The following participants were included in the study: (a) pregnant women attending the antenatal clinic within Anand district; (b) women in their first trimester of pregnancy; (c) willing to provide informed consent for participation in the study. The following participants were excluded from the study: (a) pregnant women who are not attending antenatal care within Anand district; (b) women beyond their first trimester of pregnancy; (c) inability or unwillingness to provide informed consent for participation in the study.

Following the study, pertinent data were collected using a pre-designed data collection form (demographic information including socio-demographic status, medical history, hemoglobin (Hb) data, morphological screening parameter, data on treatment, medication adherence, and outcome data). Hb levels of each participant were recorded at the end of each trimester. Participants were categorized into two groups based on the WHO guidelines: anemic (Hb < 11gm/dL) and non-anemic (Hb ≥ 11gm/dL) [9].

Additionally, the prescribed drug regimen for anemia management in both anemic and non-anemic groups was documented. The essentiality of prescribed drugs was assessed using the WHO Essential Medicines List (WHO-EML) for 2023 and the National List of Essential Medicines-2022 (NLEM-2022) [10]. Prescribed drugs were further categorized according to the FDA drug risk category.

Data analysis involved descriptive analysis and frequency distribution analysis conducted using Microsoft Excel (Microsoft Corporation, Redmond, WA). Statistical evaluation, including chi-square tests (p < 0.05), was performed using GraphPad Prism 7.0 (GraphPad Software, San Diego, CA) to ensure a comprehensive assessment of the collected data.

## Results

Prevalence of anemia

**Figure 2:**
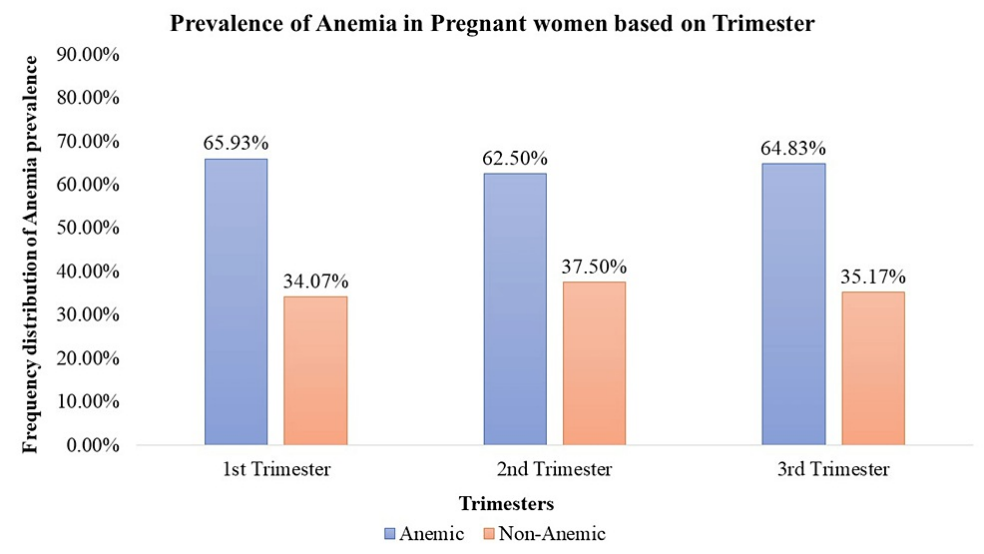
Prevalence of anemia in pregnant women based on trimester

The prevalence of anemia among 816 pregnant women was assessed across three trimesters. During the 1st trimester, 65.93% (n = 538) of pregnant women were found to be anemic, while 34.07% (n = 278) were non-anemic. In the second trimester, 62.50% (n = 510) of pregnant women were anemic, with 37.50% (n = 306) classified as non-anemic. Similarly, in the 3rd trimester, 64.83% (n = 529) of pregnant women were anemic, and 35.17% (n = 287) were non-anemic. The overall average prevalence of anemia across the three trimesters was calculated as 64.42% (n = 525.67), indicating a significant impact (p < 0.05) on the pregnant population. However, the prevalence of anemia did not show statistically significant (p > 0.05) differences across trimesters (Figure 2).

Prescribed drugs

Iron Supplementation

**Figure 3:**
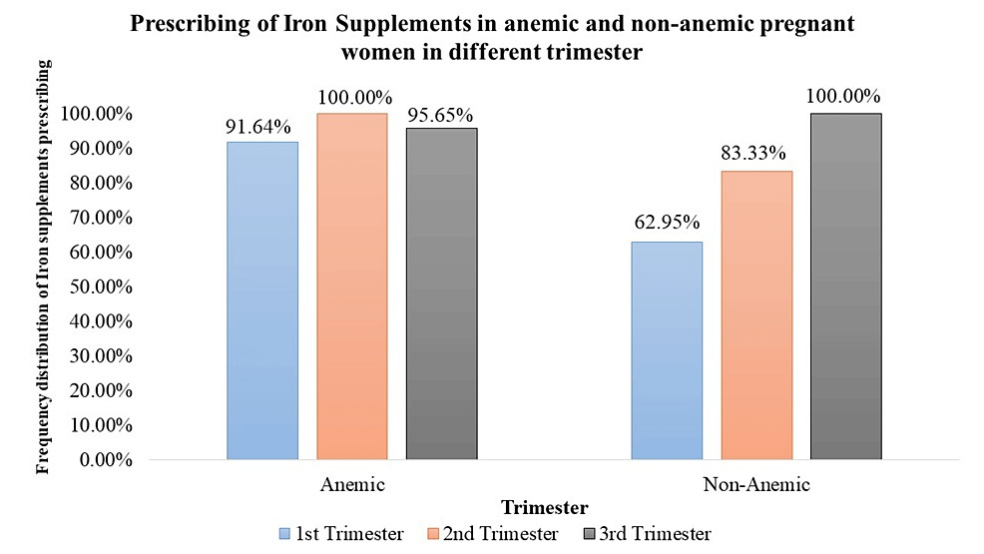
Prescription of iron supplements in anemic and non-anemic pregnant women in different trimesters

Iron supplements were significantly prescribed to both anemic and non-anemic pregnant women (p < 0.05). In the 1st trimester, 493 anemic pregnant women (91.64%) and 175 non-anemic pregnant women (62.95%) received iron supplementation. In the 2nd trimester, all anemic pregnant women (100%, n = 510) and 83.33% (n = 255) of non-anemic pregnant women received iron supplements. Similarly, in the 3rd trimester, iron supplements were prescribed to 95.65% (n = 506) of anemic pregnant women and all non-anemic pregnant women (100%, n = 287). These variations in prescription rates were statistically significant (p < 0.05) across trimesters. Iron supplements were primarily prescribed as an iron salt (Figure 3).

Folic Acid Supplementation

**Figure 4:**
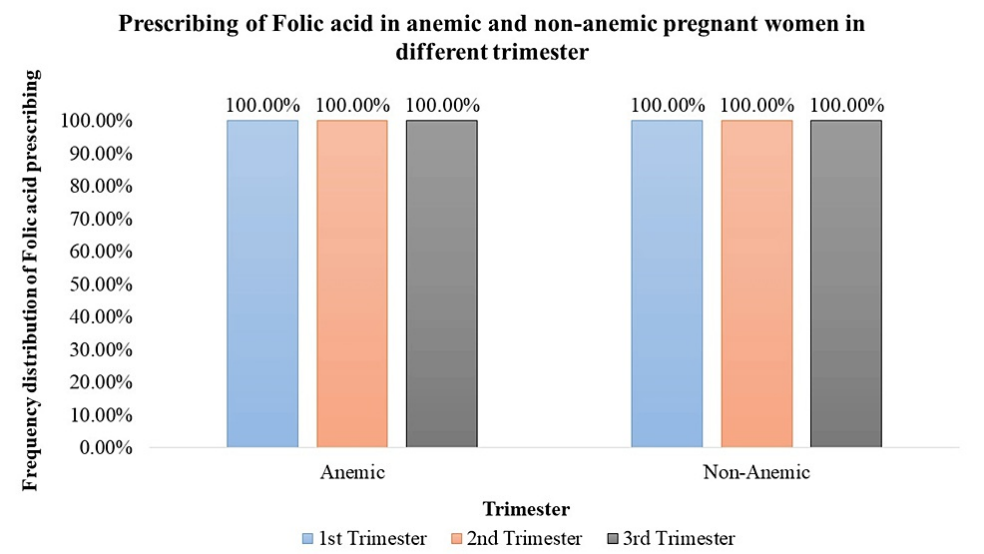
Prescription of folic acid in anemic and non-anemic pregnant women in different trimesters

Folic acid supplementation exhibited a consistent and significant trend across trimesters (p < 0.05), with both anemic and non-anemic pregnant women receiving prescriptions. Throughout all three trimesters, all pregnant women, regardless of anemia status, were prescribed folic acid. In the 1st trimester, 100% of anemic pregnant women (n = 538) and 100% of non-anemic pregnant women (n = 278) received folic acid. Similarly, in the 2nd trimester, folic acid was prescribed to 100% of anemic pregnant women (n = 510) and 100% of non-anemic pregnant women (n = 306). The trend persisted in the 3rd trimester, with 100% of anemic pregnant women (n = 529) and 100% of non-anemic pregnant women (n = 287) receiving folic acid supplementation. These prescription rates remained consistently high across all trimesters, indicating the importance and uniformity of folic acid supplementation in prenatal care (Figure 4).

Iron Sucrose Supplementation

**Figure 5:**
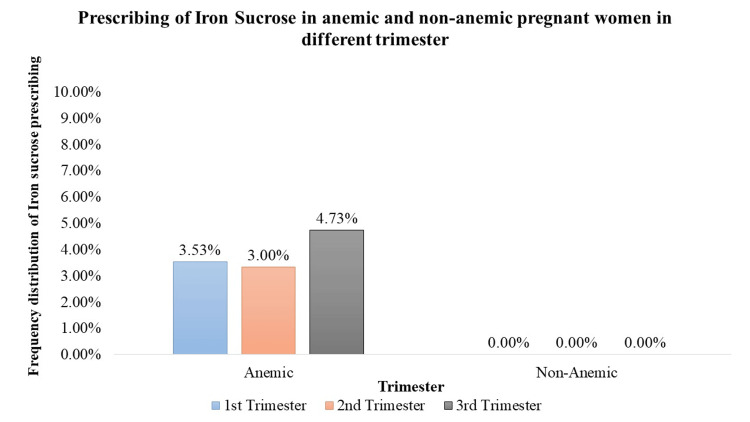
Prescription of iron sucrose in anemic and non-anemic pregnant women in different trimesters

Iron sucrose supplementation, utilized as a parenteral iron therapy, was significantly prescribed (p < 0.05) but to a lesser extent among anemic pregnant women across all trimesters. In the 1st trimester, approximately 3.53% of anemic pregnant women (n = 19) received iron sucrose supplementation. Similarly, in the 2nd trimester, 3.33% of anemic pregnant women (n = 17) were prescribed iron sucrose, and in the 3rd trimester, this percentage increased slightly to 4.73% (n = 25) of anemic pregnant women. These prescription rates indicate a more targeted use of iron sucrose, particularly in cases where oral iron supplementation may be inadequate or poorly tolerated (Figure 5).

Calcium Supplementation

**Figure 6:**
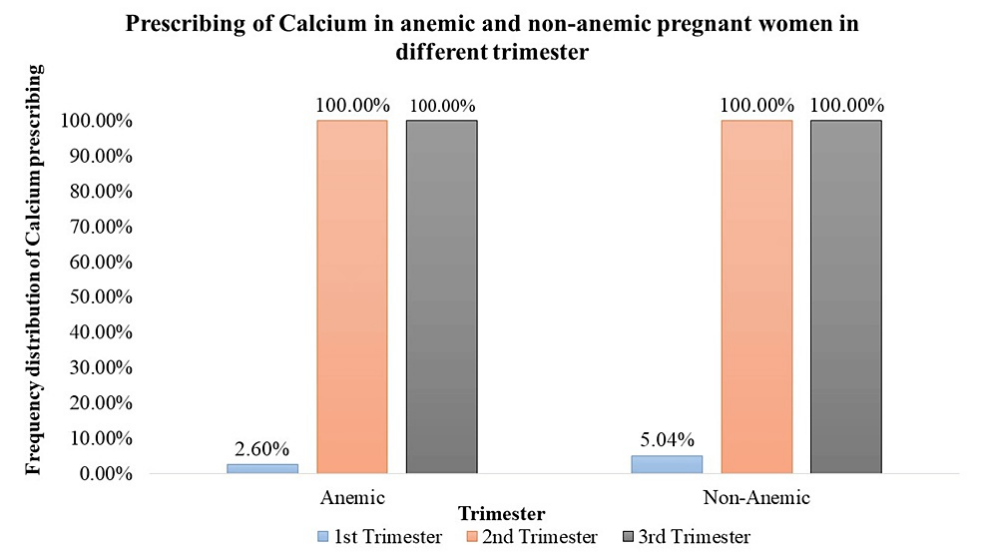
Prescription of calcium in anemic and non-anemic pregnant women in different trimesters

Calcium supplementation was prescribed to 2.60% of anemic pregnant women (n = 14) and 5.04% of non-anemic pregnant women (n = 14) during the 1st trimester. Interestingly, all pregnant women, both anemic and non-anemic, received calcium supplements during the 2nd trimester (anemic (n = 510), non-anemic (n = 306)) and 3rd trimester (anemic (n = 519), non-anemic (n = 287)), indicating universal supplementation during later stages of pregnancy. However, statistical analysis did not reveal significant differences in the prescription rates across trimesters (Figure 6).

Ascorbic Acid (Vitamin C) Supplementation

**Figure 7:**
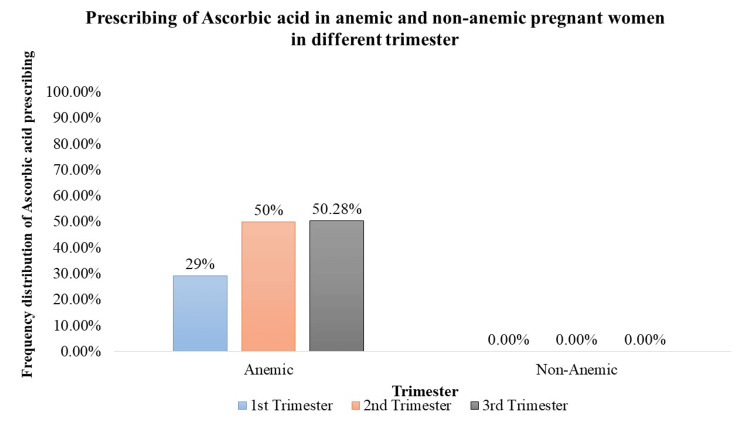
Prescription of ascorbic acid in anemic and non-anemic pregnant women in different trimesters

Ascorbic acid supplementation was significantly (p < 0.05) prescribed to anemic pregnant women during the 1st trimester (29%, n = 156), 2nd trimester (49.80%, n = 254), and 3rd trimester (50.28%, n = 266). This consistent trend underscores the importance of vitamin C supplementation in addressing anemia throughout pregnancy (Figure 7).

Multivitamin Supplementation

**Figure 8:**
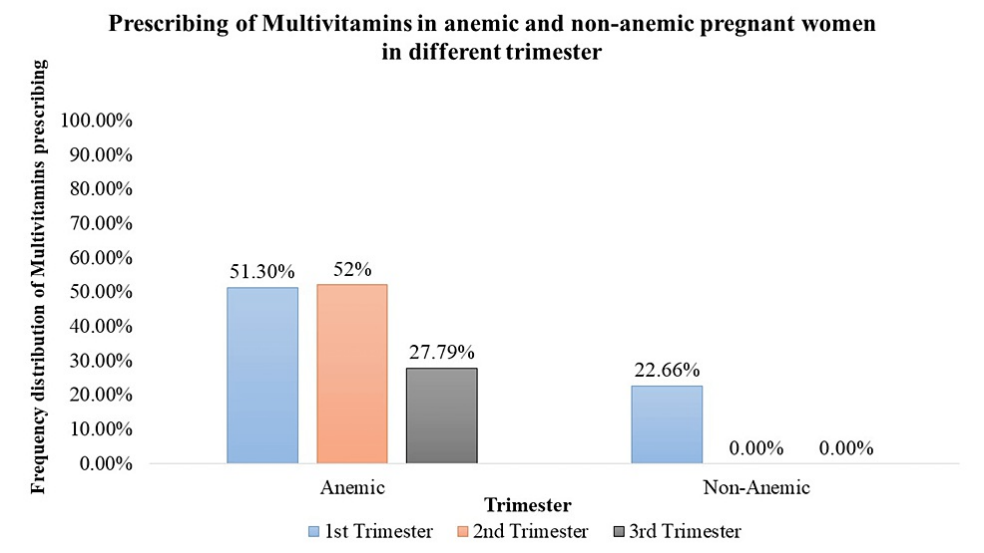
Prescription of multivitamins in anemic and non-anemic pregnant women in different trimesters

Multivitamins were significantly (p < 0.05) prescribed across all trimesters. In the 1st trimester, approximately 51.30% of anemic pregnant women (n = 276) and 22.66% of non-anemic pregnant women (n = 63) were prescribed multivitamins. Similarly, in the 2nd trimester, 52.16% of anemic pregnant women (n =266) received multivitamin prescriptions. In the 3rd trimester, 27.79% of anemic pregnant women (n = 147) were prescribed multivitamins. These findings highlight the consistent utilization of multivitamin supplementation throughout pregnancy to address various nutritional needs (Figure 8).

Essentiality and rationality

According to the WHO-EML 2023 list, oral therapies such as iron salt, folic acid, calcium, ascorbic acid, and multivitamins are considered essential for prenatal care. Conversely, iron sucrose, utilized as a parental iron therapy, is classified as non-essential. However, the NLEM-2022 list designates both oral and parental forms of iron (iron salt and iron sucrose), along with folic acid, calcium, ascorbic acid, and multivitamins, as essential.

FDA drug category

Prescribed medications were assessed using FDA drug risk categories. The majority of drugs, including iron, folic acid, ascorbic acid, calcium, and multivitamins, were classified under category A, indicating a favorable safety profile for use during pregnancy. However, iron sucrose, employed as a parenteral iron therapy, was categorized as belonging to category B, suggesting a slightly higher risk compared to oral medications.

## Discussion

This research evaluates the prevalence of anemia in 816 pregnant women throughout three trimesters and assesses the prescribing patterns of micronutrient supplements. The results indicate a substantial burden of anemia throughout pregnancy, with an overall average prevalence of 64.42%. The investigation revealed that during the 1st trimester, 65.93% of the pregnant women experienced anemia, indicating a substantial burden at the onset of pregnancy. In the 2nd trimester, there was a marginal reduction in anemia prevalence, with 62.50% of pregnant women affected. However, a subsequent rise occurred in the 3rd trimester, reaching 64.83%. These fluctuations suggest a dynamic pattern in anemia prevalence during the progression of pregnancy. The fluctuations in anemia prevalence across trimesters may be attributed to various factors, including hormonal changes, increased blood volume, and heightened demand for iron during fetal development [11]. While the lack of statistical significance in prevalence differences infers that the observed changes may fall within expected variation, it is important to thoroughly investigate potential contributing factors. Future studies could explore the interplay of nutritional factors, socio-economic status, and genetic predispositions in understanding the dynamics of anemia prevalence during pregnancy. The study also explores the prescription rates of essential micronutrient supplements, including iron, folic acid, calcium, ascorbic acid, and multivitamins, in both anemic and non-anemic pregnant women.

Iron supplementation is a cornerstone in prenatal care, crucial for preventing and treating anemia [12]. The study demonstrates a significant prescription of iron supplements to both anemic and non-anemic pregnant women. Notably, in the 1st trimester, a high percentage of anemic (91.64%) and non-anemic (62.95%) women received iron supplementation. In the 2nd and 3rd trimesters, varying but significant percentages of both anemic and non-anemic individuals were prescribed iron supplements, emphasizing the continued importance of iron support throughout pregnancy. Folic acid, essential for neural tube development [12], is consistently prescribed across all trimesters. The study highlights a significant trend, with 100% of pregnant women, both anemic and non-anemic, receiving folic acid in each trimester. Iron sucrose, employed as a parental iron therapy, is significantly prescribed but to a lower percentage of anemic pregnant women across all trimesters. This indicates a more targeted use, potentially in cases of severe anemia where oral supplementation may be insufficient. Calcium supplementation prevents pre-eclampsia, pre-term birth, and neonatal mortality (NNM), improves maternal bone mineral content, and is also vital for skeletal development, exhibiting varying prescription rates [13]. Although a small percentage of anemic and non-anemic pregnant women received calcium in the 1st trimester, the data did not reveal statistical significance. However, 100% prescription rates during the 2nd and 3rd trimesters indicate the recognition of its importance in the later stages of pregnancy. Vitamin C, known for its important role in enhancing iron absorption, is significantly prescribed to anemic pregnant women across all trimesters. In addition, it can also reduce the risk of preterm and term prelabor rupture of membranes (PROM) [14]. The varying percentages (29% to 50.28%) underscore the tailored approach to addressing iron-related concerns during different stages of pregnancy. Multivitamins, encompassing a spectrum of essential nutrients, are significantly prescribed throughout pregnancy. The study reveals varying but consistently significant percentages of anemic and non-anemic pregnant women receiving multivitamins in each trimester.

Our study, focusing on anemia prevalence among 816 pregnant women and their supplementation patterns during pregnancy, contrasts with Sappani et al.'s investigation, which examines trends in severe and moderate anemia (SMA) among women of reproductive age over 15 years in India. We found consistently high prevalence rates of anemia across trimesters among pregnant women, with an overall average prevalence of 64.42%. Our analysis revealed significant prescription patterns of various supplements, including iron and folic acid, to address anemia during pregnancy, but no statistically significant differences in prevalence across trimesters. In contrast, Sappani et al. report a decline in SMA prevalence among both pregnant and non-pregnant women over the same period, indicating potential improvements in addressing anemia among women of reproductive age overall. Both studies highlight socioeconomic factors as significant determinants of anemia and emphasize the importance of targeted interventions to improve maternal and fetal health outcomes in India [15]. The findings are also discussed in the context of the 2023 WHO-EML and the NLEM-2022.

The divergent classifications between WHO-EML 2023 and NLEM-2022 highlight the complexity of micronutrient recommendations in prenatal care. According to WHO-EML 2023, several oral therapies are deemed essential for prenatal care. This includes iron salt, folic acid, calcium, ascorbic acid (vitamin C), and multivitamins. These recommendations align with established nutritional guidelines, emphasizing the importance of these micronutrients for maternal and fetal health. Notably, iron sucrose, classified as a parental iron therapy, is deemed non-essential in this context. This classification suggests a preference for oral iron therapies in managing iron deficiencies during pregnancy. These recommendations likely stem from considerations of feasibility, cost-effectiveness, and ease of administration associated with oral supplementation. In contrast, the NLEM-2022 list designates a broader spectrum of micronutrients as essential. Both oral and parental forms of iron (iron salt and iron sucrose), along with folic acid, calcium, ascorbic acid, and multivitamins, are considered essential. This inclusion of iron sucrose as essential reflects a more comprehensive approach, recognizing the potential need for parental iron therapy in specific clinical scenarios.

The use of FDA drug risk categories provides a systematic approach to evaluate the safety of prescribed medications for anemia in pregnant women. The FDA drug risk categories play a pivotal role in regulatory decisions and guidelines. Iron, folic acid, ascorbic acid, calcium, and multivitamins, commonly prescribed for anemia in pregnant women, fall under category A. However, the categorization of iron sucrose under category B highlights a distinction in risk compared to oral medications (Table 1). It is typically prescribed in specific clinical scenarios where oral iron therapy may be impractical, ineffective, or poorly tolerated.

**Table 1:** FDA drug risk category of prescribed medications

FDA drug risk category	Medication(s)
Category A	Iron, folic acid, ascorbic acid, calcium, and multivitamins
Category B	Iron sucrose

Limitations of the study include the sample size. This study involves 816 pregnant women from a specific geographic location (Anand district). While this may be appropriate for addressing local concerns, the findings may not be generalizable to a broader population. Secondly, the study focuses on pregnant women attending the antenatal clinic during their first trimester. This may introduce selection bias as it excludes women who seek care later in pregnancy. Another limitation of the study pertains to the recording of Hb levels, which was only conducted at the end of each trimester. Anemia status could potentially fluctuate within a trimester, and more frequent measurements could provide a more accurate representation of anemia prevalence and its dynamics during pregnancy.

## Conclusions

In conclusion, the findings of this study underscore a commendable and conscientious approach to addressing anemia during pregnancy. The widespread adoption of prophylactic iron and folic acid therapy reflects a proactive stance toward mitigating the risks associated with maternal anemia. Moreover, the selective prescription of additional drugs based on individual patient needs demonstrates a tailored and patient-centered approach to care. Significantly, the absence of category X drugs, indicative of high-risk medications, from all prescriptions highlights a commitment to medication safety and underscores the cautious prescribing practices observed in the study. Furthermore, the prevalent use of cost-effective generic drugs underscores the importance of evidence-based and economically viable solutions in maternal healthcare settings. Overall, the prescription pattern observed in this study not only reflects a commitment to evidence-based practice but also emphasizes the crucial role of prophylactic measures in managing anemia during pregnancy. These findings contribute valuable insights to the broader discourse on pharmacotherapy and maternal healthcare, providing a foundation for further research and policy development in this critical area.
